# Diffusible substances from lactic acid bacterial cultures exert strong inhibitory effects on *Listeria monocytogenes* and *Salmonella enterica* serovar enteritidis in a co-culture model

**DOI:** 10.1186/s12866-017-0944-3

**Published:** 2017-02-15

**Authors:** Solomon H. Mariam, Nigus Zegeye, Abraham Aseffa, Rawleigh Howe

**Affiliations:** 1 Section of Microbiology, Aklilu Lemma Institute of Pathobiology, Addis Ababa University, P.O. Box 1176, Addis Ababa, Ethiopia; 2 Armauer Hansen Research Institute (AHRI), Addis Ababa, Ethiopia; 3 Debre Berhan University, Debre Birhan, Ethiopia

**Keywords:** Lactic acid bacteria, *L. monocytogenes*, *S*. Enteritidis, Co-culture, Inhibition, Viable but nonculturable

## Abstract

**Background:**

Food-borne infections cause huge economic and human life losses. *Listeria monocytogenes* and *Salmonella enterica* serovar Enteritidis are among the top ranking pathogens causing such losses. Control of such infections is hampered by persistent contamination of foods and food-processing environments, resistance of pathogens to sanitizing agents, existence of heterogeneous populations of pathogens (including culturable and viable but non-culturable cells) within the same food items, and inability to detect all such pathogens by culture-based methods. Modern methods such as flow cytometry allow analyses of cells at the single cell level within a short time and enable better and faster detection of such pathogens and distinctions between live and dead cells. Such methods should be complemented by control strategies including the use of beneficial bacteria that produce metabolites capable of inhibiting food-borne pathogens. In this study, broth cultures of lactic acid bacteria (LAB) isolated from fermented milk were tested for production of substances capable of inhibiting *L. monocytogenes* and *S.* Enteritidis in co-culture with LAB by assessment of colony-forming units (CFU) and live:dead cell populations by flow cytometry.

**Results:**

The LAB isolates belonged to the species *Lactococcus lactis, Enterococcus faecalis and Enterococcus faecium*. Some LAB were effective in inhibition. Plating indicated up to 99% reduction in CFU from co-cultures compared to control cultures. Most of the bacteria in both cultures were in the viable but non-culturable state. The flow data showed that there were significantly higher dead cell numbers in co-cultures than in control cultures, indicating that such killing was caused by diffusible substances produced by the LAB cultures.

**Conclusion:**

This study showed that metabolites from selected local LAB species can be used to significantly reduce pathogen load. However, conditions of use and application need to be further investigated and optimized for large-scale utilization.

**Electronic supplementary material:**

The online version of this article (doi:10.1186/s12866-017-0944-3) contains supplementary material, which is available to authorized users.

## Background

*Listeria monocytogenes* is a notorious food-borne pathogen that is capable of switching between saprophytic and intracellular parasitic life styles [1]. Moreover, it resists ordinary disinfectants and survives exposure to various stresses and food preservation methods such as low pH, low temperatures and the presence of salt, thus, enabling the pathogen to survive on surfaces in food processing facilities, in the human gut, during refrigeration and in marine waters [1–4]. Traditional culture-based methods are not always adequate to detect the presence of the pathogen due to their inherent limitations. A variety of enrichrnent and selective culture media that enable distinction between pathogenic and non-pathogenic *Listeria* species have been formulated for isolation of *Listeria* spp. from foods. The sensitivity and specificity of these various media can be affected by the type of food matrix; however, these culture-based methods and molecular detection methods enable enhanced identification and enumeration of *L. monocytogenes* [5, 6]. Moreover, methodologies combining phenotypic and molecular analyses provide the necessary information on the prevalence, contamination level, antibiotic resistance profiles, genetic relatedness and ecological preferences of *Listeria* spp. from various food sources [7, 8].

The infectious dose and subsequent sequel of infection with *L. monocytogenes* may be dependent on the specific strain (e.g., food or clinical isolate), and age and immune status of the host [9–11]. As an intracellular pathogen, it can cross the intestinal, blood-brain and feto-placental barriers and cause gastroenteritis, septicemia, meningitis, encephalitis and abortion or stillbirth of neonates [12–15]. The same groups at high risk to listeriosis are also at risk to acquiring salmonellosis. *Salmonella enterica* serovar Enteritidis (*S*. Enteritidis) is a major cause of food-borne salmonellosis in humans worldwide, although clinical isolates can vary in their degree of virulence [16–18].

Microbial analyses of foods and biological samples are traditionally carried out by direct plating on agar media. Food spoilage bacteria often persist in the foods, including when under stress, without being amenable to detection by culture. This leads to false negative results or underestimation of bacterial load. The failure to detect spoilage bacteria by culture might be due to entry of part of the bacterial population into a viable but non-culturable (VBNC) state [19]. Thus, the existence in the same food item of mixed populations of bacteria in different physiological states (including culturable and VBNC bacteria) limits the application of traditional methods because such methods are limited in their capacities to resolve heterogeneous populations to the single cell level. Conventional PCR is not generally useful for detection of viable organisms [20]. Modern methods such as flow cytometry allow investigations at the single cell level, including distinction between live and dead cells, and enable analysis of large populations of samples within a short period of time [21, 22]. For these purposes, membrane-penetrating dyes that stain both live and dead cells (e.g., SYTO dyes) as well as membrane-impermeable dyes (e.g., propidium iodide) that stain dead cells are used. Such distinctions between live (culturable and VBNC) and dead bacteria can have several applications (e.g., in evaluation of the effectiveness of disinfection processes, in food microbiology, in environmental microbiology). Three major criteria used to distinguish among culturable, VBNC and dead bacteria are culturability, metabolic activity and membrane integrity [20, 23].

The VBNC state is induced by stress (such as unfavorable temperatures or pH, deprivation of oxygen or nutrients, high or low osmotic concentrations, and exposure to commonly used food preservatives) [19, 24, 25]. VBNC state cells are considered to be in a persistent state, which allows them to survive and then revive upon the return of favorable conditions, and are also more resistant to drugs [19, 26, 27].

The oldest food preservation methods involve fermentation. Fermentation is driven by a community of beneficial microbes, notably lactic acid bacteria (LAB). Microbial interactions during food fermentations involve antagonism and competitions between LAB species and pathogens when contaminating pathogens are present. The LAB antagonize against such pathogens by various mechanisms (e.g., by production of organic acids, hydrogen peroxide and bacteriocins [28, 29].

*L. monocytogenes* and other pathogens not only pose significant threat to food safety but may also display resistance to antibiotics. Outbreaks of food-borne infections are frequently reported globally. All these call for alternative methods that can be applied either alone or in combination with other food preservation methods to counter contamination and pathogen load. LAB are prime candidates in the search for such alternatives and may be used in various ways (e.g., direct challenge with live LAB, use of their metabolic products or fermentate from LAB cultures) [29].

Studies on possible antagonistic effects of LAB in co-culture with pathogenic bacteria as targets appear to be rare. The objective of this study was the determination of inhibitory activities of secreted substances from selected LAB species in co-culture with *L. monocytogenes* or *S.* Enteritidis. The growth pattern of the pathogens was monitored at defined time intervals by both plate counts and flow cytometry.

## Methods

### Bacteria

#### Isolation of LAB

LAB were isolated from a bovine milk sample that was being served to customers in a local cafeteria. Media used for isolation of LAB included MRS agar, M17 agar, and PCA agar (Oxoid, UK) plates. Milk was allowed to ferment for 3 days at room temperature. A sample of the fermented milk was serially diluted in sterile PBS buffer, pH 7.2 and plated on the above media. Single colonies were picked at random from each plate (15 in total), inoculated into 10 mL MRS broth and incubated for 18 h at 37 °C at either aerobic or anaerobic condition to get samples of each isolate for long-term storage (OXOID AnaeroGen AN0035ACE jar and gasket [OXOID, UK]) were used for anaerobic incubations).

#### Target organisms

*L. monocytogenes* (ATCC 19115) and *S.* Enteritidis (ATCC 13076) were used as target organisms. *L. monocytogenes* was grown in tryptic soy broth (TSB), *S.* Enteritidis was grown in Luria-Bertani (LB) broth. Cells were pelleted and resuspended in TSB or LB broth containing 15% glycerol, aliquoted and stored frozen. A fresh aliquot was taken for each experiment.

#### Selection of LAB for inhibitory effect

Filter-sterilized cell-free supernatants that were prepared from broth cultures of each identified LAB isolate were preliminarily tested separately and in combinations for inhibitory effect on broth cultures of target organisms to which the supernatants had been added at 1:8 or 1:4 proportion. Effect was assessed by changes in optical density readings at 600 nm using a spectrophotometer. Among the 15 isolates tested, 10 isolates failed to retard the growth of *L. monocytogenes* and *S.* Enteritidis during this preliminary test for inhibitory effect while the remaining 5 isolates that showed promise were selected for further studies in co-cultures.

#### Identification of LAB

MRS agar was streaked with samples from the MRS broth cultures for identification by standard cultural and biochemical tests [30, 31]. Tests conducted for presumptive phenotypic identification of the isolates included Gram staining, colony morphology, catalase and nitrate reduction tests, and motility test.

**Fig. 1:**
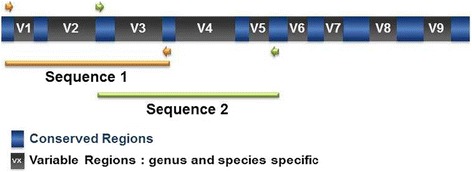
Hypervariable regions V1 to V5 (sequence 1 and sequence 2) of 16S rRNA gene amplified with universal primers for species level identification of LAB isolates used in this study

The LAB isolates were further identified based on their 16S rRNA gene sequences by amplification of extracted DNA. The hypervariable regions encompassing regions V1-V5 (Fig. 1) were amplified and sequenced. Phylogenetic trees showing the relatedness of the isolates to known LAB species were constructed. Sequences were compared to the sequences in public (NCBI) database using BLAST. A negative control was run to verify the contamination-free state of the samples and *E. coli* DNA was included as an internal positive quality control.

#### Culture and data collection

Costar Transwell polycarbonate permeable membrane supports (No. 3419) (diameter of 75 mm, pore size of 0.4 μm and pore density of 1 × 10^8^ pores/cm^2^) were used. The Transwell has an upper and a lower chamber, with volume capacities of 9 mL and 13 mL respectively. The upper chamber has three slits for pipetting access to the lower chamber. For *L. monocytogenes*, two types of cultures were set-up: (i) control cultures: 9 mL of plain MRS broth was transferred into the upper chambers and 13 mL of TSB inoculated with *L. monocytogenes* was transferred into the lower chambers; (ii) co-cultures: 9 mL of MRS broth prewarmed to 37 °C was inoculated with a single colony each of the 5 LAB species and transferred into the upper chambers and 13 mL of TSB inoculated with *L. monocytogenes* was transferred into the lower chambers. For *S.* Enteritidis, the same control culture and co-culture set-ups were made except that *L. monocytogenes* was replaced by *S.* Enteritidis and TSB was replaced by LB broth. The number of *L. monocytogenes* and *S.* Enteritidis cells inoculated into the 13 mL TSB or LB broth was 5.20x10^3^ mL^−1^ and 5.15 × 10^3^ mL^−1^ respectively. The number of LAB in all co-culture upper chambers was 1.19x10^5^ mL^−1^.

Once these cultures were set up, the Transwell were incubated at 37 °C for 12 h and then transferred to and maintained at 4 °C for the remainder of the experimental period (22 days) (to assess the survival of *L. monocytogenes* and *S.* Enteritidis in the co-culture conditions). Samples were withdrawn at various time points, starting from just after inoculation, for both quantitation of colony-forming units (CFU) and flow cytometry (FCM) analysis.

#### Flow cytometry analysis

**Fig. 2:**
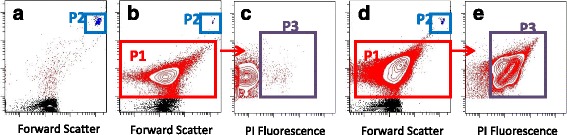
Gating strategy to identify and quantitate total and dead bacteria. Cultured *L.monocytogenes* or *S.* Enteritidis were either untreated (Panels **b**, **c**) or heat-killed (**d**, **e**), diluted in PBS, and counting beads and propidium iodide (Panels **b** through **e**) added prior to acquisition. Bacteria were defined as events present within gate P1, and were distinct from events in the PBS buffer + beads control (Panel **a**). Counting beads were utilized as an internal control to normalize acquisition volume between samples, and were identified in gate P2. Dead cells were defined in gate P3 as propidium iodide-fluorescent positive events among P1-gated cells

Optimization experiments to determine the proper time for propidium iodide (PI) uptake were conducted by using live and heat-killed cells of *L. monocytogenes* and *S.* Enteritidis. Fresh aliquots were grown to mid-log phase in TSB or LB broth. Then, PI was added to live and heat-killed cells and the percentage live and dead cells analyzed by FCM at 5 min intervals for 50 min. For FCM, 30 μL samples from control cultures and co-cultures were withdrawn and three successive 10-fold dilutions were made in pH 7.2 filtered phosphate buffer saline (PBS) to a final volume of 300 μL. Propidium iodide (Sigma, P4170) was used to identify dead cells. One μL of a 1 mg/mL stock solution of PI was added to each diluted sample along with 20 μL of flow cytometry counting beads (BD 51-90-9001229) diluted 1 in 6 in PBS, agitated several times and analysed by FCM in a BD FACSCanto II flow cytometer. The forward and side light scatter PMT voltages were adjusted to visualize small sized events and the light scatter threshold minimized. Bacteria exhibited light scatter properties substantially above background scatter of PBS alone, but significantly lower than the flow cytometry beads. Events with intermediate light scatter properties corresponding to bacteria and events with high scatter (cytometer beads) were independently gated (gates P1 and P2, respectively) and total counts within each gate were determined. Events expressing PI among P1- gated bacteria were identified (gate P3) and were used to determine the percentage of dead bacteria (Fig. 2).

Total bacteria per sample were calculated as observed events within P1 times the total beads per sample divided by the beads observed in P2. Total dead cells were determined by multiplying total cell number by the fraction of dead cells. Live bacteria were defined as total bacteria less dead bacteria. Finally, the number of VBNC cells was calculated as live bacteria minus culturable bacteria. Percentage inhibition of growth was calculated as 100 x (1-(live bacteria in the experimental sample/live bacteria in the control sample)). The experiments were conducted three independent times following prior optimizations.

#### Test for resuscitation of VBNC state cells

To assess if resuscitation of samples from co-cultures occurs when sub-cultured under optimum conditions, samples were withdrawn and inoculated into 5 mL fresh TSB (*L. monocytogenes*) or LB broth (*S.* Enteritidis) within ordinary culture tubes (sub-culture 1). Immediately after inoculation into sub-culture 1, samples were plated on tryptic soy agar (TSA) (*L. monocytogenes*) or LB agar (*S.* Enteritidis) and the inoculated 5 mL culture tubes were incubated at 37 °C. After 24 h of incubation, samples from sub-culture 1 were plated again on TSA or LB agar and incubated. Furthermore, following incubation for 24 h of sub-culture 1, samples from sub-culture 1 were sub-cultured into 5 mL fresh broth (sub-culture 2) and incubated, followed by plating after 24 h of incubation.

#### Partial characterization of antimicrobial substances

In a separate set-up, LAB were cultured in the upper chamber, with the lower chamber of the Transwell containing un-inoculated TSB or LB broth and incubated at 37 °C for 12 h (most of the inhibitory effect is exerted within the first 12 h in co-culture) with or without LAB-inoculated MRS broth in the upper chamber. The TSB or LB broth was recovered, filtered through a 0.4 μm pore size filter and portions were either heat-treated (100 °C, 1 h) or untreated. and either pH-adjusted to 6.5 or unadjusted (the pH before adjustment was 4.6 ± 0.2), and catalase-treated or untreated. These treatments were similarly repeated for supernatants from the LAB-inoculated MRS broth of the upper chamber. *L. monocytogenes* or *S.* Enteritidis were inoculated into the heat-treated or untreated filtrates at 1:100, 1:10 or 1:1 ratio (filtrate:fresh TSB or LB broth, v/v) and incubated at 37 °C for 24 h.

### Statistical analysis

For both control cultures and co-cultures, both CFU assays and FCM analyses were conducted in parallel. The CFU values were calculated per mL basis and log-transformed. The ‘*t*’ test was used to determine if significant differences existed between control culture and co-culture CFUs using GraphPad Prism v. 6 (LaJolla, CA). A *P* value ≤ 0.05 was considered to indicate a significant difference.

## Results

### Identification of LAB isolates

The selected LAB isolates were all Gram-positive cocci, catalase- and nitrate reductase-negative, non-motile and formed pairs or chains. They were presumptively considered to be LAB.

The phylogenetic tree species-level identification of the LAB isolates indicated the isolates were *Lactococcus lactis* (isolates S2 and S6), *Enterococcus faecalis* (isolate S3) and *Enterococcus faecium* (isolates S11 and S15). The partial 16S rRNA gene sequences were obtained from the amplified fragments.

**Fig. 3:**
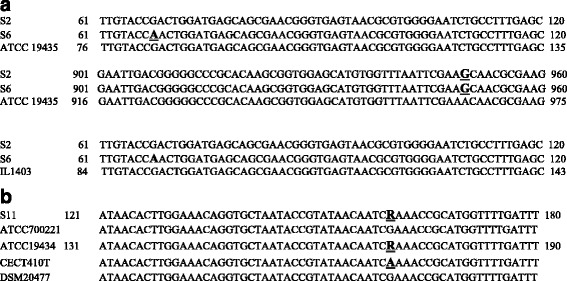
**a** 16S rRNA nucleotide sequence alignment of *Lactococcus lactis* isolates S2 and S6 with the sequence of *Lactococcus lactis* subsp. lactic ATCC 19435 (upper and middle panels) and with *Lactococcus lactis* subsp. lactis IL1403 (lower panel) (nucleotide differences are highlighted). **b** 16S rRNA nucleotide sequence alignment of *Enterococcus faecium* isolate S11 with the sequence of *Enterococcus faecium* type strains ATCC 700221, ATCC 19434, CECT 410 T and DSM 20477 (nucleotide differences are highlighted)

The nucleotide sequence alignment showed that there was a 100% sequence identity between the 16S rRNA gene sequence of S2 and the sequence of *Lactococcus lactis* subsp. *lactis*, strain IL1403 16S ribosomal RNA gene but showed 99% sequence identity with that of *Lactococcus lactis* subsp. *lactis* ATCC 19435 (synonym: *Lactococcus lactis* subsp. lactis JCM 5805 T) [32] with nucleotide G instead of A (Fig. 3a). The isolate S6 possessed nucleotide A instead of G at position 68 relative to both of ATCC 19435 and IL1403, while both isolates S2 and S6 exhibited a change of A to G relative to that of ATCC 19435 at position 950 (Fig. 3a). The sequence of S2 also showed 100% identity to several other *L. lactis* strains in the database. In any case, the lowest identity to any *L. lactis* sequence was 99%.

The sequence of the 16S rRNA gene of isolate S3, identified here as *Enterococcus faecalis*, exhibited 99% sequence identity to those of *Enterococcus faecalis* strains ATCC 19433, ATCC 29212, CECT481T and DSM 20478 T. It has nucleotide ‘Y’ instead of T at position 633 as BLAST analysis showed (indicating the presence of two clones in the sample) (data not shown).

Isolate S11was identified as *Enterococcus faecium*. Its sequences showed 99% identity to the 16S rRNA gene sequences of *E. faecium* strains ATCC 700221, ATCC 19434, CECT410T and DSM 20477. S11 differed from all these strains in having only an R in place of G at position 70 (data not shown). However, S11 resembled the type strain ATCC 19434 in having an R at position 160 (indicating a G or A at that position), while strains ATCC 700221 and DSM20477 both had a G and CECT410T had an A at that same position (Fig. 3b). The same pattern was found for S15.

### CFU data from plate cultures

#### L. monocytogenes

**Fig. 4:**
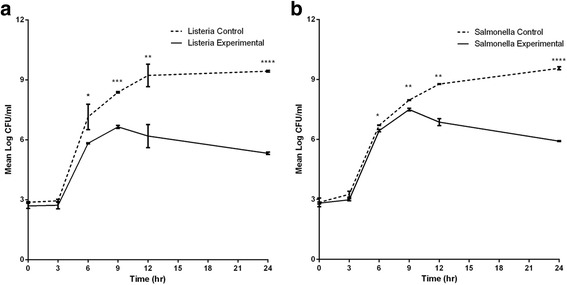
Log_10_CFU values from control cultures and co-cultures of *L. monocytogenes* (**a**) and *S.* Enteritidis (**b**) plated at 3 h intervals from Transwell control cultures and co-cultures. Hour 0 equals CFU values immediately after inoculation of cultures. The results at each time point are the means ± standard deviations for 3–4 replicate cultures. Asterisks indicate significantly higher CFU numbers of control cultures than those of co-cultures (experimental). *, *P* ≤ 0.05; **, *P* ≤ 0.01; ***, *P* ≤ 0.001; ****, *P* ≤ 0.0001

There were no significant differences in CFU between the control cultures and co-cultures for up to 3 h of incubation (Fig. 4a). Following a lag phase of 3 h, the control cultures grew exponentially until the 6th hr and continued to grow at slightly slower rate for a further 6 h (to > 9 Log CFU ml^−1^). The control cultures continued to grow at a much slower rate until the 24th hr and then stabilized (Fig. 4a and data not shown). Exponential growth was also seen in the co-cultures until the 6th hr with further slowed growth until the 9th hr (to 6.65 Log CFU mL^−1^). Thereafter, it continued to decrease during the remaining period of the experiment (Fig. 4a and data not shown). The CFUs from the control cultures were significantly higher than those from the co-cultures at 6, 9, 12 and 24 h (Fig. 4a). This difference was maintained during the entire experimental period. In addition, from co-cultures, colony formation on agar was delayed by 2–3 days, the sizes of the colonies were much smaller and the numbers much fewer (1.8 Log reduction) than colonies from control cultures (Additional file 1: Figure S1).

At the end of the experiments, the fluid from the control culture was densely turbid (tube 2 in Additional file 1: Figure S2) while that from the co-culture showed no turbidity at all (tube 4 in Additional file 1: Figure S2), indicating strong inhibition in the co-cultures.

#### *S.* Enteritidis

There were no significant differences in CFU between the control cultures and co-cultures during the initial 3 h (Fig. 4b). However, CFUs from the co-cultures became significantly lower than those from the control cultures starting after 6 h of incubation (Fig. 4b). This difference was maintained during the entire experimental period (Additional file 1: Table S1). The CFU from the co-cultures increased during the later days of 15 and 22 (Additional file 1: Table S1). This increase was also reflected in the turbidity of the fluid withdrawn from the co-culture bottom chamber at the end of the experiment (tube 8 in Additional file 1: Figure S2).

### Resuscitation

Attempts were made to test the resuscitation of cells from co-cultures of *L. monocytogenes* and *S.* Enteritidis by making successive sub-cultures (in broth and then on agar) as described in Methods. Initially, it was observed that colonies from samples of co-cultures were fewer and much smaller than colonies from control cultures. The colonies that resulted from 24 h-incubated samples of sub-culture 1 were exactly as seen before for samples from the co-cultures (i.e., fewer and smaller than those from the control cultures). However, the colonies that resulted from 24 h-incubated samples from sub-culture 2 were equal, in both numbers and sizes of colonies, to those from the control cultures, with no visible differences between them (data not shown).

Tests for resuscitation of cells from *S.* Enteritidis co-cultures gave similar results to those obtained for *L. monocytogenes*.

### Assessment of bacterial cell killing by flow cytometry

Thirty-five minutes was found to give the maximum PI uptake by dead cells, with no more increase in dead cells despite further prolonged incubation. Furthermore, live cells did not show significantly increased PI uptake as time increased from 0 to 35 min. There were no differences in dead cell (P3) events, as revealed by the FCM analysis, between heat-killed and live cells in the absence of PI staining. Initially, it was also proven that there were no populations in the dead cells region when non-PI-stained cells (from control cultures or co-cultures) as well as heat-killed or live cells were analyzed.

#### L. monocytogenes

The percentage of dead cells in the co-cultures on day 0 (i.e., in an hr after culture set-up) were not higher than those from the corresponding control cultures. However, on days 8, 15 and 22, they were all significantly higher than those from the corresponding control cultures (*P* < 0.05) (Table 1A).

**Table 1:** Dead cell populations in control cultures and co-cultures of *L. monocytogenes* (A) and *S.* Enteritidis (B) by FCM analysis

Bacteria	Day	Culture	Mean % dead cells	95% CI of mean	*P* value
A.					
	0	C	0.48	0.23–0.73	
	0	E	0.95	0.33–1.57	*P* > 0.10
	8	C	3.03	1.20–4.86	
	8	E	12.63^a^	5.55–19.71	*P* < 0.05
	15	C	4.13	−1.22–9.48	
	15	E	28.2^a^	15.18–41.21	*P* < 0.05
	22	C	1.70	1.59–1.81	
	22	E	9.73^a^	5.44–14.02	*P* < 0.05
B.					
	0	C	0.68	0.02–1.34	
	0	E	1.80	1.62–1.98	*P* > 0.10
	8	C	14.0	10.11–17.89	
	8	E	93.27^b^	88.0–98.53	*P* < 0.005
	15	C	33.4	31.08–35.72	
	15	E	90.0^b^	87.95–92.05	*P* < 0.005
	22	C	63.47	60.07–66.87	
	22	E	91.0^b^	87.75–94.25	*P* < 0.005

Assessed using the *t* test. ^a^and^b^ indicate significantly higher dead cell population in co-cultures versus the corresponding control cultures of *L. monocytogenes* and *S.* Enteritidis respectively at the same day. Day 0 refers to time about 1 h after culture set-up. Mean is the percentage of total dead cells among three replicate assay tubes that are PI-stained. CI = confidence interval, C = control cultures, E = co-cultures

**Fig. 5:**
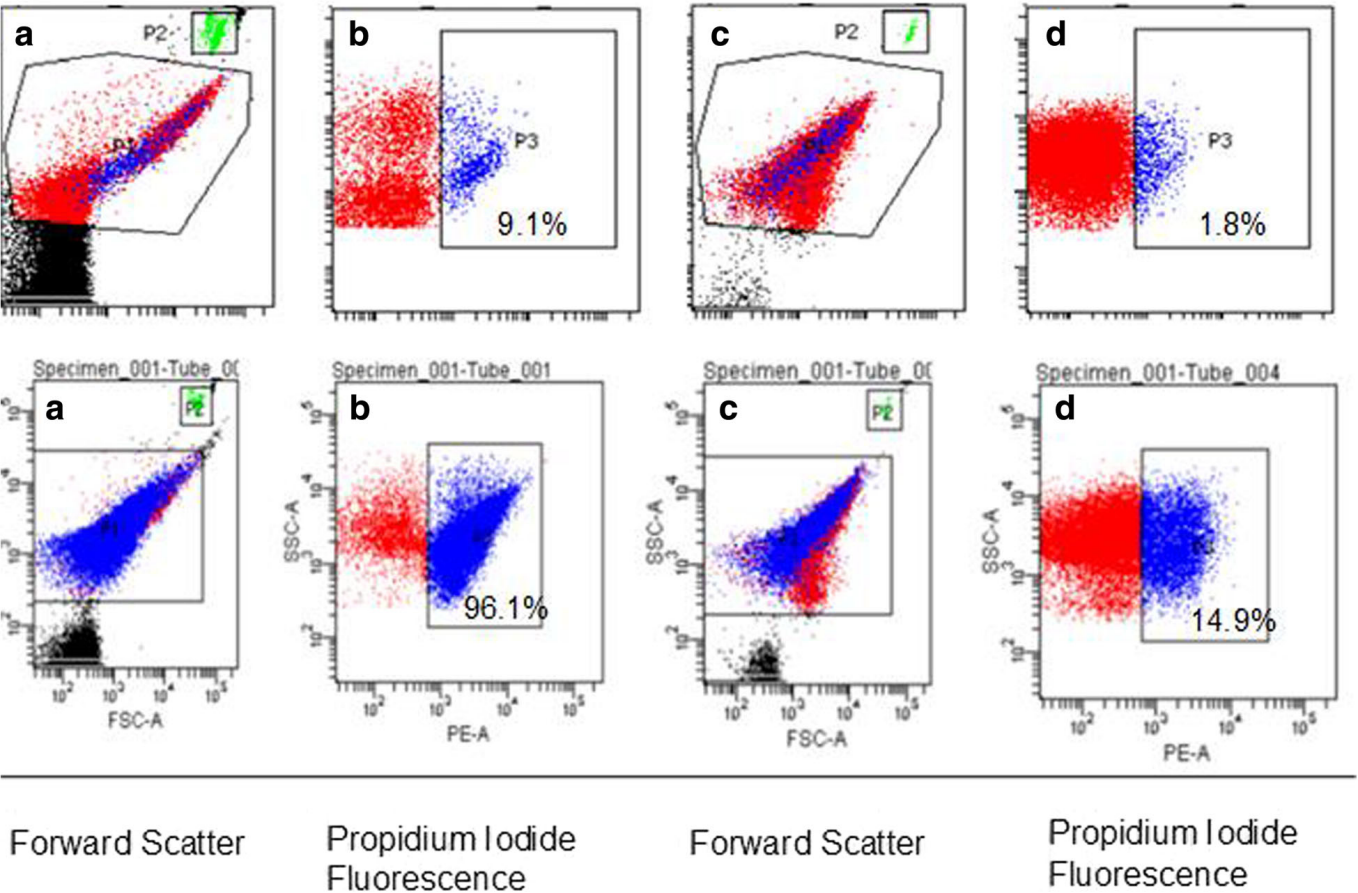
Representative results from flow cytometry analyses of dead cell staining of co-culture and control cultures. Upper panel: *L.monocytogenes* were either co-cultured with LAB (**a**, **b**) or cultured without LAB (control, **c**, **d**), and evaluated for dead cells (identified within P3) by PI staining as defined in Fig. 1. Dead cells (within P3) comprised 9.1% of P1- gated *L.monocytogenes* from co-cultures (**b**), whereas only 1.8% of P1-gated *L.monocytogenes* cells were identified as dead cells in control cultures (**d**). Lower panel: *S.* Enteritidis were either co-cultured with LAB (**a**, **b**) or cultured without LAB (control, **c**, **d**), and evaluated for dead cells (identified within P3) by PI staining as defined in Fig. 1. Dead cells (within P3) comprised 91% of P1-gated *S.* Enteritidis from co-cultures (**b**), whereas 60% of P1-gated *S.* Enteritidis cells were identified as dead cells in control cultures (**d**)

Initially, more than 99% of the cells in both the control cultures and co-cultures were in the VBNC state (Additional file 1: Table S1A). The percentage of VBNC cells from the control cultures decreased to 68% by day 8 (probably earlier) and then increased to > 90% thereafter. The percentage of cells from the co-cultures that were in the VBNC state was consistently >90%, but modestly decreased by day 22. The percentage of dead cells in the co-cultures on days 8 and 22, as the FCM analyses reported, were similar. So were the percentage of dead cells in the control cultures on days 8 and 22. The upper panel of Fig. 5 shows data from day 22. The percent inhibition of cells in co-cultures relative to those from control cultures at the same day was consistently > 99% (data not shown). At this time point, there were five-fold more dead cells from co-cultures relative to those from control cultures (Fig. 5 upper panel b and d respectively).

#### *S.* Enteritidis

The percentage of dead cells from the co-cultures were significantly higher than those from the respective control cultures on days 8, 15 and 22 (Table 1B) (*P* < 0.005). During the latter days of 8, 15 and 22, the *S.* Enteritidis control cultures also showed increasingly higher percentages of dead cells, but still significantly less than those from co-cultures (Table 1B).

The percentage of VBNC state cells from co-cultures was >99% on day 0 but decreased to 74% and to 61% on days 8 and 15 respectively and then increased to 87% on day 22 (Additional file 1: Table S1B). The decrease in percent VBNC cells in co-culture on days 8 and 15 was accompanied by an increase in percent dead cells when compared to the percent dead cells on day 0. The percent culturable cells form co-culture also increased on days 8 and 15 (Additional file 1: Table S1B). A decrease in percent VBNC in control cultures was accompanied by an increase in percent culturable cells and vice versa (Additional file 1: Table S1B). The percentage of dead cells in the co-cultures on days 8 and 22 were ≥ 90%. While the percentage of dead cells in the control cultures was 13% on day 8, it increased to >60% on day 22 (Fig. 5 lower panel and Additional file 1: Table S1). The percent inhibition in co-culture was > 99% on days 8 and 15 but decreased to 65% on day 22 (data not shown). The FCM analysis reported that 96% of cells from co-cultures were dead while 15% of cells from control cultures were dead on day 8 (Fig. 5, lower panel) and this agreed with the calculated values (Additional file 1: Table S1B).

### Effect of heat treatment, pH adjustment and treatment with catalase

*L. monocytogenes* or *S.* Enteritidis inoculated into the heat-treated or untreated filtrate at 1:100, 1:10 or 1:1 ratio (filtrate:fresh TSB or LB broth, v/v) and incubated for 24 h were inhibited at the 1:1 ratio (but not at the 1:100 and 1:10), indicating the inhibitory substances were transferred into the lower chamber and were heat-resistant. Similarly, both heat-treated and untreated filtrate from the upper chamber of LAB-inoculated MRS broth was effective in inhibition at the 1:1 ratio. Adjustment of the pH to a higher pH of ~6.5 abolished the inhibitory effect of the portions that showed inhibitory effect before adjustment. Catalase treatment did not abolish inhibitory activity indicating non-involvement of hydrogen peroxide in the inhibition (data not shown).

## Discussion

Here, we set out to determine the pattern of growth of target organisms in co-culture with LAB in a manner that would reasonably allow us to attribute any growth inhibitory or lethal effects to substances secreted by the LAB cultures. Highly-significant reductions in CFU as well as increases in dead cell populations were obtained in co-cultures compared to control cultures.

To study the effect of LAB on the growth of *L. monocytogenes* and *S.* Enteritidis, direct competition experiments in mixed cultures were not considered in this work for two reasons: (i) the recommended media required for optimum growth of the bacterial species are different, (ii) assessment of the effect of LAB by the assays used here (CFU quantitation and FCM analysis) would be difficult because established differential characteristics (e.g., phenotypic selectable marker(s) are not known. Thus, the co-culture model was chosen because it allows the bacteria to be cultured in their own media for assessment of numbers of CFUs and total live and dead cells and attribute any growth-inhibitory effect to substances secreted from the LAB cultures.

The mechanisms proposed to play roles in inhibition of pathogens by LAB include competitive exclusion, competition for nutrients, production of organic acids, and production of antimicrobial substances [29, 33]. The first two can be reasonably ruled out as having any roles in these experiments, narrowing the role to antimicrobial diffusible substances (i.e., bacteriocins or other substances). However, it would be inappropriate to attribute all killing to the diffusible substances. Significantly higher dead cell populations in co-cultures would justify attribution of inhibitory effects to the diffusible substances. The action of the diffusible substances was bactericidal, as confirmed by the FCM analysis (although resistance to the substances may have developed or the bacterial cells may have entered into a state unresponsive to the substances, since not all cells were killed).

The total population of cells in the cultures could be divided into three different physiological states; i.e., culturable, VBNC, and dead cells. In these populations, total cell count exceeds that of live cells (culturable and VBNC combined). The CFU data can be misleading since they suggested that ≥ 99% of the cells in co-cultures were killed. In reality, however, the percentage of dead cells is much less than that as revealed by the FCM analyses. This exemplifies the limitation of culture in detecting the presence of VBNC state cells.

Since the percent VBNC value is a subset of the overall live cells counted by FCM, the decrease in percent VBNC during the latter days (relative to those of day 0) could be due to the decrease in the total live cells during those latter days, which can occur due to combined effects of several stress factors. The decrease in percent VBNC in control cultures (e.g., day 8 *L. monocytogenes* control) could indicate more cells exited the VBNC state and became culturable, but during prolonged stressful storage at 4 °C, more cells again entered the VBNC state. As cells exit the VBNC state, the culturable count increases and as cells re-enter the VBNC state, the culturable count decreases. We postulate that a dynamic process exists in which those cells that become culturable may follow one of three trajectories due to the combined effects of several stress factors (and probably due to cell-to-cell signaling as well): remain culturable, re-enter the VBNC state, or become extinct (which is manifested as increased dead cell population).

Resuscitation of VBNC cells of various bacteria has been achieved by temperature up-shift [34], supplementation with pyruvate [35], growth in embryonated eggs [36], using growth factors [37], suspension together with amoebae [38], and co-culture with eukaryotic cells [39, 40]. Here, we were able to restore culturability of presumably VBNC state co-culture cells by simple removal of the low temperature stress factor and sub-culturing in fresh un-supplemented broth, with no differences in the size, number and rate of growth of resulting colonies of co-culture and control culture cells. Small size may be one feature of VBNC state cells exposed to nutrient limitation and other stresses [19, 41, 42] and this feature was repeatedly observed here in colonies of cells drawn from co-cultures. The finding of higher growth rate and reduced generation times for *L. monocytogenes* as well as *S.* Enteritidis in both the control cultures and co-cultures compared to that of broth-grown culture is also indicative of resuscitation of the cells, since, under the ideal conditions of broth-grown culture, growth rate should be higher and generation time shorter. This burst in growth rates and generation times of the co-cultures was of course gradually reversed past the 6 h mark of incubation. However, we cannot rule out the possibility of re-growth of a minority population of culturable cells.

The effect of the diffusible substances on the target bacteria might be further enhanced if continuous culture systems (for continuous production of the diffusible substances). Moreover, such effect could possibly be enhanced if pathogenic Gram-negative bacteria were subjected to osmotic shock, exposure to low pH or agents that cause release of the lipopolysaccharide [33]. The storage conditions of the co-cultures used here (4 °C) and the nutrient exhaustion [43] during the prolonged incubations can be considered limitations on the continuous production of the diffusible substances from the LAB cultures.

Several modes of application can be considered (e.g., direct inoculation of the selected LAB species, addition of purified active substances into fermenting foods, replacement of uncharacterized starter cultures with defined strains, inactivation or reduction of emergence of antibiotic-resistant bacteria by combined application of the active substances with antibiotics [33, 44, 45]. Further characterizations and studies with respect to potential side effects (e.g., presence of virulence factors), strain-specific effects, influence of gut microbiota, diet, presence of morbidities, and suitability to the target population will be needed to produce scientifically-validated strains [46].

The limitations of this study include the lack of clinical isolates of LAB and additional type strains of *L. monocytogenes* and *S*. Enteritidis to conduct the tests described herein and the use of one cytometry dye. Furthermore, we did not isolate and identify the active inhibitory substances.

## Conclusions

This model may be modified and combined with other methods such as dialysis and chromatography for purification of the active metabolites. Appropriate modifications of this model may be used to test its potential to reduce pathogen load and decontaminate sea foods such as shrimp and oysters that are prone to contamination by pathogens such as *Vibrio* spp. Some studies indicate that using lactic acid and other organic extracts can be useful adjuncts to control food-borne pathogens and enhance food safety [47–50]. The purified metabolites can be more applicable, especially in acidic environments such as the gut, where some LAB species may be unable to resist. The food forms (milieu) should be taken into consideration in assessing the effects of the substances [51]. The model and assay methods used here allow for both analysis of presence of pathogens and inhibition of pathogens. Since inhibition of pathogens is one of the desirable properties in probiotic species, this model will serve as a method to screen additional LAB species for inhibitory activities, whether with or without additional probiotic benefits. Selected local LAB species have potential to reduce pathogen loads in foods, especially for pathogens that require high infectious doses to establish infection. The possible existence of VBNC state pathogens (with capability for in vivo resuscitation and cause infection/disease) should always be considered when inhibition is achieved. More detailed studies are needed to elucidate the relationships between growth phases of bacteria in different environments and their possible existence in, and exit from, the VBNC state as well as their potential for virulence.

## Additional file

Additional file 1: Table S1. Comparison of calculated culturable counts (CFUs), live and dead cell populations and VBNC cells from control cultures and co-cultures of *L. monocytogenes* (A) and S. Enteritidis (B). C = control cultures, E = co-cultures, CC = culturable count. **Figure S1.** Comparative colony sizes of *L. monocytogenes* plate cultures from Transwell control culture (A) and co-culture (B). (A) 1 µL of a 10^–3^ dilution plated, (B) 1 µL of a 10^–1^ dilution plated. (A) after 24 hrs of incubation of plated culture, (B) after 48 hrs of incubation of plated culture. Very similar patterns were observed for S. Enteritidis control culture and co-culture samples. **Figure S2.** Turbidity of fluid from upper and lower chambers of control cultures and co-cultures. Fluid was withdrawn from upper and lower chambers of *L. monocytogenes* (tubes 1-4) or *S*. Enteritidis (tubes 5-8) at the end of the experiments. Tubes 1 and 2: fluid from upper and lower chambers respectivey, of *L. monocytogenes* control culture. Tubes 3 and 4: fluid from upper and lower chambers respectivey, of *L. monocytogenes* co-culture. Tubes 5 and 6: fluid from upper and lower chambers respectivey, of *S*. Enteritidis control culture. Tubesd 7 and 8: fluid from upper and lower chambers respectivey, of *S*. Enteritidis co-culture. (DOCX 243 kb)

## Abbreviations

CFU: Colony-forming units
FCM: Flow cytometry
LAB: Lactic acid bacteria
LB: Luria-Bertani
PBS: Phosphate buffer saline
PI: Propidium iodide
TSA: Tryptic soy agar
TSB: Trptyic soy broth
VBNC: Viable but nonculturable
