# Supplementary material for: Diffusible substances from lactic acid bacterial cultures exert strong inhibitory effects on Listeria monocytogenes and Salmonella enterica serovar enteritidis in a co-culture model
Source: BMC Microbiol. 2017 Feb 15;17:35. doi: 10.1186/s12866-017-0944-3 (PMC5312424; doi:10.1186/s12866-017-0944-3)
Supplement: Additional file 1: Table S1. — Comparison of calculated culturable counts (CFUs), live and dead cell populations and VBNC cells from control cultures and co-cultures of L. monocytogenes (A) and S. Enteritidis (B). C = control cultures, E = co-cultures, CC = culturable count. Figure S1. Comparative colony sizes of L. monocytogenes plate cultures from Transwell control culture (A) and co-culture (B). (A) 1 µL of a 10–3 dilution plated, (B) 1 µL of a 10–1 dilution plated. (A) after 24 hrs of incubation of plated culture, (B) after 48 hrs of incubation of plated culture. Very similar patterns were observed for S. Enteritidis control culture and co-culture samples. Figure S2. Turbidity of fluid from upper and lower chambers of control cultures and co-cultures. Fluid was withdrawn from upper and lower chambers of L. monocytogenes (tubes 1-4) or S. Enteritidis (tubes 5-8) at the end of the experiments. Tubes 1 and 2: fluid from upper and lower chambers respectivey, of L. monocytogenes control culture. Tubes 3 and 4: fluid from upper and lower chambers respectivey, of L. monocytogenes co-culture. Tubes 5 and 6: fluid from upper and lower chambers respectivey, of S. Enteritidis control culture. Tubesd 7 and 8: fluid from upper and lower chambers respectivey, of S. Enteritidis co-culture. (DOCX 243 kb) [file 12866_2017_944_MOESM1_ESM.docx]

Table S1. Comparison of calculated culturable counts (CFUs), live and dead cell populations and VBNC cells from control cultures and co-cultures of *L. monocytogenes* (A) and *S.* Enteritidis (B). C = control cultures, E = co-cultures, CC = culturable count. **A**

Day Sample Total cells gated No of dead cells(%) Live cells(% ) CC (% of live cells) Putative VBNC

No % of live cells

0 C 2.2x10^7^ 1.95x10^5^(0.9) 2.18x10^7^(99) 8.27x10^3^(0.04) 2.17x10^7^ 99.5

E 1.85x10^7^ 3.3x10^5^(1.8) 1.82x10^7^(98) 1.25x10^4^(0.07) 1.82x10^7^ 99.9

8 C 9.15x10^9^ 3.02x10^8^(3.3) 8.85x10^9^(96.7) 2.83x10^9^(32) 6.02x10^9^ 68.0

E 2.26x10^7^ 3.74x10^6^(16.5) 1.88x10^5^(83.4) 8.02x10^5^(4.2) 1.8x10^7^ 95.7

15 C 6.64x10^9^ 9.39x10^7^(1.4) 6.54x10^9^(98.5) 3.42x10^8^(5.2) 6.2x10^9^ 94.7

E 1.21x10^7^ 4.08x10^6^(33.7) 8.02x10^6^(66.3) 2.62x10^5^(3.3) 7.76x10^6^ 96.7

22 C 9.83x10^9^ 1.63x10^8^(1.1) 9.67x10^9^(98.3) 2.86x10^8^(2.9) 9.4x10^9^ 97.0

E 1.63x10^7^ 1.98x10^6^(12.1) 1.43x10^7^(87.7) 2.52x10^6^(17.6) 1.18x10^7^ 82.4

**B**

Day Sample Total cells gated No of dead cells(%) Live cells (%) CC (% of live cells) Putative VBNC

No % of live cells

0 C 1.94x10^7^ 1.64x10^5^(0.8) 1.92x10^7^(99) 1.51x10^4^(0.08) 1.92x10^7^ 99.9

E 4.03x10^7^ 1.24x10^6^(3.1) 3.90x10^7^(96.9) 2.47x10^4^(0.06) 3.9x10^7^ 99.9

8 C 1.92x10^9^ 2.59x10^8^(13.5) 1.66x10^9^(86.5) 8.90x10^8^(53.6) 7.70x10^8^ 46.4

E 1.87x10^8^ 1.80x10^8^(96.2) 7.0x10^6^(3.7) 1.80x10^6^(25.7) 5.20x10^6^ 74.3

15 C 1.36x10^9^ 4.46x10^8^(32.8) 9.14x10^8^(67.2) 2.51x10^8^(27.5) 6.63x10^8^ 72.5

E 5.21x10^8^ 4.68x10^8^(89.8) 5.30x10^7^(10.0) 2.07x10^7^(39) 3.27x10^7^ 60.9

22 C 1.37x10^9^ 8.98x10^8^(65.5) 4.72x10^8^(34.4) 4.15x10^8^(87.9) 1.22x10^8^ 12.1

E 1.37x10^9^ 1.23x10^9^(89.8) 1.40x10^8^(10.2) 1.80x10^7^(12.9) 1.22x10^8^ 87.1

A
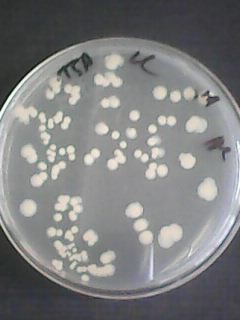

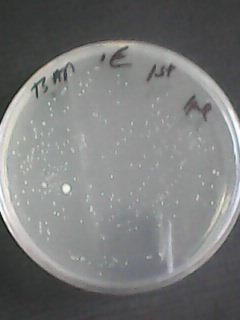
B

Figure S1. Supplementary material. Comparative colony sizes of *L. monocytogenes* plate cultures from Transwell control culture (A) and co-culture (B). (A) 1 μL of a 10^−3^ dilution plated, (B) 1 μL of a 10^−1^dilution plated. (A) after 24 hrs of incubation of plated culture, (B) after 48 hrs of incubation of plated culture. Very similar patterns were observed for *S.* Enteritidis control culture and co-culture samples.


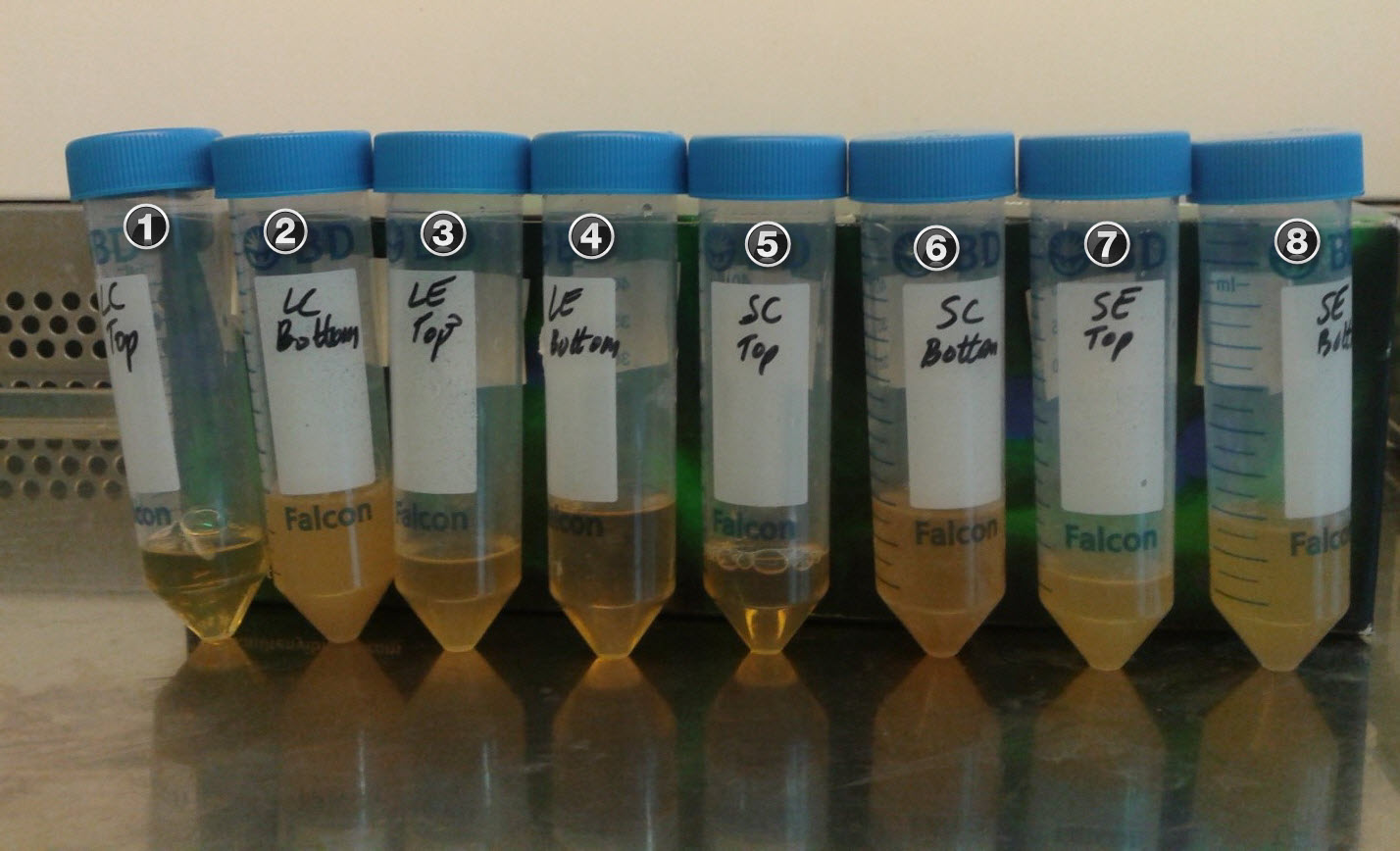


Figure S2. Suppelmentary material. Suppelmentary material. Fluid was withdrawn from upper and lower chambers of *L. monocytogenes* (tubes 1-4) or *S.* Enteritidis (tubes 5-8) at the end of the experiments. Tubes 1 and 2: fluid from upper and lower chambers respectivey, of *L. monocytogenes* control culture. Tubes 3 and 4: fluid from upper and lower chambers respectivey, of *L. monocytogenes* co-culture. Tubes 5 and 6: fluid from upper and lower chambers respectivey, of *S.* Enteritidis control culture. Tubesd 7 and 8: fluid from upper and lower chambers respectivey, of *S.* Enteritidis co-culture.
